# Evaluation of reserpine-induced fibromyalgia in mice: A comparative behavioral, neurochemical, and histological assessment of two doses

**DOI:** 10.1016/j.ibneur.2024.11.002

**Published:** 2024-11-09

**Authors:** Hanin Abdulbaset AboTaleb, Emad A. Hindi, Gamal S. Abd El-Aziz, Hani A. Alturkistani, Mervat M. Halawani, Mona Ali Al-Thepyani, Badrah S. Alghamdi

**Affiliations:** a Department of Physiology, Faculty of Medicine, King Abdulaziz University, Jeddah 21589, Saudi Arabia; b Department of Clinical Anatomy, Faculty of Medicine, King Abdulaziz University, Jeddah 22252, Saudi Arabia; c Department of Chemistry, College of Sciences & Arts, King Abdulaziz University, Rabigh 21911, Saudi Arabia; d Neuroscience and Geroscience Research Unit, King Fahd Medical Research Center, King Abdulaziz University, Jeddah 21589, Saudi Arabia

**Keywords:** Fibromyalgia, Reserpine, Depression, Histology, Hippocampus, Pain, Serotonin

## Abstract

Fibromyalgia (FM) is a chronic pain disorder characterised by widespread musculoskeletal pain, fatigue, and cognitive disturbances. Due to its complex and poorly understood pathophysiology, reliable animal models are essential for research. Reserpine is commonly used to induce FM in animals, effectively replicating key symptoms such as widespread pain, hypersensitivity, and mood disturbances. However, the varying doses used in studies and the often unclear rationale for dose selection have led to a lack of standardisation for this model. In this study, we investigated the effects of commonly used reserpine doses (0.25 and 0.5 mg/kg) to induce FM in mice. Following three consecutive days of subcutaneous reserpine injections, we conducted detailed behavioural assessments and microscopic examinations of tissue changes. We also compared the effects of the two reserpine doses on serotonin, norepinephrine, glutamate, interleukin (IL)-1β, and tumour necrosis factor (TNF)-α levels in the brain and spinal cord. The behavioural assessment demonstrated that both doses of reserpine had nearly the same effect relatively early after administration. Histological examination revealed neurotoxic effects of reserpine, particularly in the hippocampus and thalamus, which was more obvious at day 11 with the 0.5 mg/kg dose. Furthermore, this dose significantly altered all neurotransmitter levels compared with the control group. While the effect of the 0.25 mg/kg dose on neurotransmitter levels was limited, it altered the IL-1β and TNF-α levels in the brain and spinal cord. Our findings indicate that injecting reserpine at 0.25 or 0.5 mg/kg for three consecutive days is effective in inducing FM-like pain in mice, characterised by mechanical and thermal hypersensitivity, along with associated depression and motor deficits. However, the 0.5 mg/kg dose is optimal for inducing persistent neurochemical alterations and histopathological changes, lasting up to 10 days following the initial injection.

## 1 Introduction

Fibromyalgia (FM) is a chronic widespread muscular pain condition associated in most cases with general fatigue, soft tissue tenderness, depression, and impaired cognitive functions (Theoharides et al., 2015, Theoharides et al., 2019). FM affects 2 %-8 % of adults aged 20–60 years, predominantly women (Branco et al., 2010). Despite its prevalence, the aetiology of FM is still a matter of debate and not fully understood. Central sensitisation, descending pain inhibitory pathways dysfunction, and depletion of biogenic monoamines are the most accepted theories behind its pathogenesis (Becker and Schweinhardt, 2012, Millan, 1999, Theoharides et al., 2015, Yoshimura and Furue, 2006). Changes in the number, diameter, and function of small nerve fibres were also reported in the patient with FM (Doppler, Rittner, Deckart, & Sommer, 2015). Animal models of FM are useful to understand the potential causes of FM, to investigate the underlying mechanisms, and to develop a definitive treatment that could stop and reverse the pathogenesis of this disease. While it is not that easy to replicate the symptoms of FM in animals due to its multifaceted nature, several methods have been used to establish FM-like symptoms in rodents, including the acid saline-induced pain model (Sluka et al., 2003, Yokoyama et al., 2007), the hyperalgesia priming model (Dina, Green, & Levine, 2008), fatigue-enhanced muscle pain (Yokoyama, Lisi, Moore, & Sluka, 2007), the cold-stress model (Nishiyori et al., 2011), the sound stress model (Khasar, Dina, Green, & Levine, 2009), and the sub-chronic swim stress model (Suarez-Roca, Leal, Silva, Pinerua-Shuhaibar, & Quintero, 2008). However, those approaches pose some challenges as they should lack tissue injury and correlate with widespread FM pain and associated co-morbidities, that is, depression, anxiety, and fatigue (DeSantana, da Cruz, & Sluka, 2013).

In 2009, Nagakura et al. established as a putative rodent model of FM by using reserpine. Since then, this model has been widely used to study FM (Nagakura et al., 2009). Reserpine is an indole alkaloid and a monoamine-depleting agent that reduces serotonin, dopamine, and norepinephrine (NE) in several brain regions (Khasar et al., 2009). It is a competitive, high-affinity inhibitor of vesicular monoamine transporter (VMAT-1 and VMAT-2). Blocking VMAT-2 decreases the uptake of intracellular monoamines into presynaptic vesicles, which prevents further release of monoamines into the synapse, causing monoamine depletion in nerve terminals (Gasnier, 2000, Yaffe et al., 2018). Due to its notable mechanism of action, reserpine has been used to induce several neurological disorders in animals besides FM, such as progressive depression (Ikram & Haleem, 2017), Parkinson's dementia (Ikram & Haleem, 2019), and tardive dyskinesia (Patil, Hiray, & Kasture, 2012). It has been found that repeated systemic administration of reserpine leads to long-lasting general muscle and cutaneous hyperalgesia that is sustained for at least one week in rodents and not reduced by nonsteroidal anti-inflammatory drugs (de Souza et al., 2014). Repeated reserpine administration to rodents was also found to cause common co-morbid symptoms of FM, including depression and anxiety (Qian, Zhong, Lu, & Zhang, 2023).

Previous studies have used varying doses of reserpine to induce FM in mice, resulting in a lack of standardisation. Some studies have successfully induced FM and its comorbidities in mice using a dose of 0.25 mg/kg administered subcutaneously over three consecutive days (de Souza et al., 2014, Klein et al., 2014, Peres Klein et al., 2016). Others have increased the dose to 0.5 mg/kg (Singh, Kaur, Singh, & Bhatti, 2021), and some have even used up to 1 mg/kg (Brusco et al., 2021; Fischer et al., 2020; Xu et al., 2013) with the same duration and route of administration. However, the overall condition of the mice deteriorated with a dose of 1 mg/kg (Nagakura et al., 2018). Despite the common use of reserpine, a consensus on the optimal dose for inducing FM-like symptoms remains elusive, with inconsistent findings related to motor activity, depression, and anxiety (Brum et al., 2020; Sałat & Furgała-Wojas, 2021; Singh, Kaur, Garg, Singh, & Bhatti, 2020). Therefore, the primary aim of this study was to comprehensively evaluate the differential effects of two commonly utilised safe doses of reserpine, 0.25 and 0.5 mg/kg, to establish a standardised model of FM induction in mice. We systematically assessed sensory, motor, depressive, anxiety-related, and cognitive behaviours following reserpine administration, as well as its effect on related biochemical and histopathological changes. The optimal reserpine dose should reliably induce FM and its associated comorbidities while minimising unwanted toxic effects and mortality. We hypothesise that the 0.5 mg/kg dose yields more pronounced FM-like symptoms and associated comorbidities, achieving an effective balance between efficacy and safety. Our study could enhance the reproducibility of FM-related research and contribute to the development of more targeted therapeutic strategies.

## 2 Materials and methods

### 2.1 Mice

The experiments involved a total of 106 male Swiss albino mice, weighing between 30 and 40 g and aged 9–10 weeks. These mice were sourced from the King Fahad Medical Research Centre in Jeddah, Saudi Arabia. Each clear, transparent polycarbonate cage housed a maximum of five mice, providing ample space and comfort. The straw bedding was changed regularly. The mice had unrestricted access to food and water and were maintained under a standard 12-hour photoperiod at 23 ± 2ºC. To ensure they were well-adapted, the mice were acclimatised to the laboratory environment for 4 days before beginning the experiments. Behavioural evaluations were performed between 9:00 a.m. and 2:00 p.m. The entire study was conducted in strict adherence to the ethical standards set by the Biomedical Ethics Committee of King Abdulaziz University (Approval Number: 236–24). The research protocol followed the guidelines of the Animal Care and Use Committee (ACUC) at the Animal House Unit, King Fahd Medical Research Centre, Jeddah, Saudi Arabia.

### 2.2 FM-like model

FM-like symptoms were induced in mice via subcutaneous reserpine injections, according to a method described in the literature (Nagakura et al., 2018). For this, 0.25 or 0.5 mg/kg reserpine was injected into the mice once a day for three consecutive days; the volume was 6.3 ml/kg. Reserpine (98 % purity, Acros Organics – Fisher Scientific) was prepared by diluting it to the desired concentration using a 0.5 % (v/v) glacial acetic acid solution in distilled water.

### 2.3 Treatment and assessment strategy

**Fig. 1:**
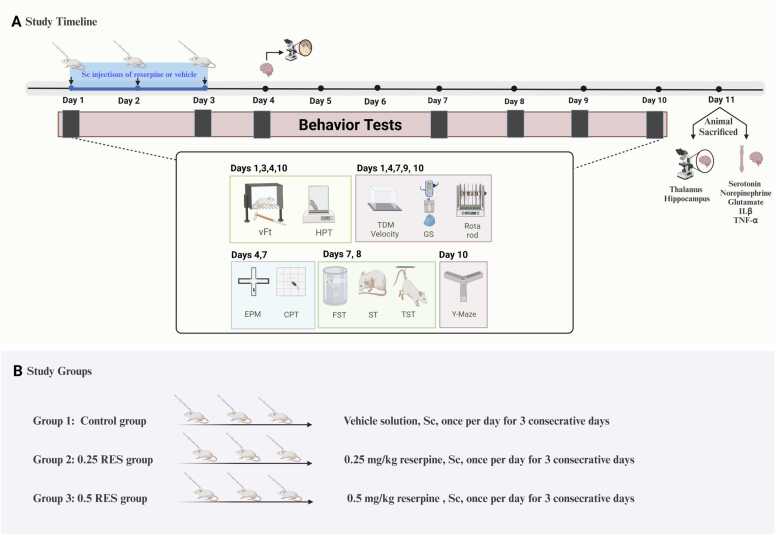
Schematic representation of the overall design and timeline of the study. (A) The total duration of the study was 11 days. On the first 3 days, reserpine was administrated by subcutaneous injections. Behavioral tests were performed on days 1, 3, 4, 7, 8, 9, and 10. The brain tissues were collected from the mice on days 4 and 11 for histopathological examination. For biochemical analysis, brain and spinal cord samples were collected on day 11. (B) Study groups, and their administrated reagents. *Abbreviations:* vFt, von Frey test; HPT, hot plate test; TDM, total distance moved; CPT, central preference test; EPM, elevated plus Maze test; GS, grip strength test; FST, forced swimming test; TST, tail suspension test; ST, splash test. *Created with BioRender.com*.

The mice were randomly divided into three groups: the vehicle control group (control), the 0.25 mg/kg reserpine (0.25 RES) group, and the 0.5 mg/kg reserpine (0.5 RES) group. The control group received subcutaneous injections of a vehicle solution (0.5 % glacial acetic acid in distilled water). The 0.25 RES group and 0.5 RES group received subcutaneous injections of reserpine at a concentration of 0.04 and 0.08 mg/ml, respectively. Each mouse received a single daily injection of vehicle or reserpine administrated into the loose skin around the neck and shoulder area, with a maximum volume of 0.25 ml, for three consecutive days. The study spanned a total of 11 days, beginning with 3 days of reserpine injections, and followed by an evaluation period. Behaviour was assessed at various time points, as depicted in Fig. 1. For the sensory and depression tests, each group of mice was used twice and once respectively to avoid any residual effects of one test on another. In motor tests that required repeated-measures analysis, the same mice were subjected to each test. Brains were collected from three mice in each group on day 4 of the study for the first histological examination. On day 11, the brains and spinal cords of the remaining mice were collected for further analysis.

### 2.4 Weight measurement

The mice were weighed every three days of the study between 09:00 and 10:00 a.m. The percentage change in body weight was calculated as

Change in body weight (%) =

$$\frac{\mathrm{Recorded time point weight}-\mathrm{Original weight}}{\mathrm{Original weight}\;}\;\times \;100.$$

In the above equation, the original weight was the weight measured before the first injection of reserpine, and the recorded time point weight was the weight recorded on the specific day.

### 2.5 Assessment of pain threshold and pain-related behaviour

#### 2.5.1 Von Frey test

Mechanical allodynia thresholds were measured using Von Frey filaments through the up-down paradigm according to a method described in the literature (Gonzalez-Cano et al., 2018). The Aesthesio® set of filaments (UGO-37450–275, Ugo Basile, Italy), which range in stiffness from 0.04 to 4 g, were used. Briefly, each mouse was placed individually in clear a plexiglass box on an elevated mesh platform (90 × 38 cm, with a mesh-like open grid of square holes ∼5 × 5 mm) for acclimation. After a 45-minute acclimation period, the examiner applied the filaments to the plantar surface of the mouse’s hind paws. Positive responses were recorded when the mouse lifted its paws, marked as (X), while no response within 5 seconds was marked as (O). The stimulation started with 0.6 g and continued for five consecutive positive responses (assigned a score of 0.04), five consecutive negative responses (assigned a score of 4), or until completion of four readings following the first different response. The paw withdrawal threshold, representing the amount of force required to elicit a response, was measured in grams using a threshold calculator website designed by Christensen et al. (Christensen et al., 2020). A significant decrease in the paw withdrawal threshold compared with the control group indicated the presence of mechanical allodynia (Klein et al., 2014).

#### 2.5.2 Hot plate test

A hot plate apparatus (Ugo Basile) was used to assess the thermal hyperalgesia in mice, as described previously (Hunskaar, Berge, & Hole, 1986). Each mouse was placed on a heated metal plate maintained at 55 ± 0.1°C (Sałat & Furgała-Wojas, 2021), enclosed by a clear acrylic cylinder. The latency to heat response, indicated by flicking, hind paw licking, or jumping, was recorded, with a cut-off of 30 seconds to prevent tissue damage. To mitigate the influence of learning behaviour as a confounding factor (Deuis, Dvorakova, & Vetter, 2017), each group of mice was used only once across the four required test time points (days 1, 3, 4, and 10 following the first reserpine injection).

#### 2.5.3 Spontaneous pain-related behaviour

The Grimace Scale subjectively assesses pain intensity based on an animal’s facial expressions (Whittaker, Liu, & Barker, 2021). Two mice were randomly selected from each group to be photographed at the required time points (days 1, 2, 3, 4, and 10 following the first reserpine injection). The mice were placed in a transplant acrylic animal-enclosure box (9 cm high × 5 cm wide) and acclimated for 30 minutes. A continuous front photo was then taken for each mouse to evaluate the Grimace Scale parameters: orbital tightening, nose and cheek bulge, ear position, and whisker change (Langford et al., 2010).

### 2.6 Measurement of motor behaviour

#### 2.6.1 Open-field test

On days 1, 4, and 9 following the first reserpine injection, each mouse was placed in the centre of an acrylic box arena (45 cm × 45 cm × 34 cm) and allowed to move freely for 3 minutes in a sound-attenuated room, under low-intensity light. The movement of the mice, including the total distance moved (in cm) and the velocity (in cm/s), was tracked and recorded using the EthoVision XT8A system (Noldus Information Technology, Wageningen, the Netherlands) (Abo Taleb and Alghamdi, 2020; Alzahrani et al., 2022).

#### 2.6.2 Grip strength test

The grip strength test is commonly used to assess the effects of various drugs on motor performance (Takeshita et al., 2017). The muscle strength of each mouse was evaluated on days 1, 4, and 10 following the first reserpine injection using a conventional automated grip strength meter (Columbus Instruments, Columbus, OH, USA). During the test, the experimenter held the mouse by its tail, allowing it to grasp the metal bar of the grip strength meter with its forelimbs. The mouse was then gently pulled until it released its grip. The device recorded the maximum muscle strength in gram-force (gf). Three measurements were taken for each mouse, with at least 1 minute between measurements, to calculate the average muscle strength (Brusco et al., 2021).

#### 2.6.3 Rotarod test

The effects of different doses of reserpine on fatigue and muscle coordination was assessed using the rotarod test, which is also recommended for assessing nociception in rodents (Altarifi, Alsalem, & Mustafa, 2019). On day 7 following the first reserpine injection, each mouse was trained on the rotarod at a speed of 9 rpm until it could remain on the rotating apparatus for 30 seconds without falling. One hour later, its ability to remain on the rotarod at a fixed speed of 20 rpm was recorded for up to 300 seconds (Dagnino et al., 2019, Nascimento et al., 2014). This procedure was repeated for a total of three trials, with a 10-minute interval between the trials, and the average performance was calculated.

### 2.7 Assessing of depressive-related behaviour

#### 2.7.1 Splash test

Anhedonia, one of the major symptoms of depression (Serretti, 2023), was assessed using the splash test to measure anhedonia-like behaviour following reserpine administration. In this test, self-cleaning performance was measured by squirting 300 μl of a 10 % sucrose solution onto each mouse’s dorsal coat. Two minutes after the application, the number of grooming episodes was recorded over 5 minutes (Martins et al., 2022). The test was conducted on day 7 following the first reserpine administration.

#### 2.7.2 Forced swimming test

The forced swimming test is used to identify depressive-like behaviour in experimental animals (Yankelevitch-Yahav, Franko, Huly, & Doron, 2015). This test was employed to measure depression in mice following reserpine administration, according to an established method (Sałat et al., 2015). Each mouse was placed in a separate glass cylinder (10 cm in diameter × 20 cm high) filled with water maintained at 23–25°C to a depth of 14 cm and allowed to swim for 6 minutes. The immobility of the mice during the last 4 minutes was recorded (Sałat & Furgała-Wojas, 2021). Immobility was defined as the absence of paw movements except those necessary to keep the head above water (Singh, Kaur, Singh, et al., 2021). The test was conducted on day 8 following the first reserpine injection.

#### 2.7.3 Tail suspension test

The tail suspension test was used to assess depression-related behaviour in mice (Cryan, Mombereau, & Vassout, 2005). Following a previously described protocol, each mouse was suspended by its tail approximately 50 cm above the bench using adhesive tape affixed to a horizontal wire. The suspension lasted for 6 minutes, and the total immobile time was recorded during the last 4 minutes. The mouse was considered immobile when it ceased struggling and remained motionless to overcome the unnatural position (Alqurashi et al., 2022). The test was conducted on day 8 following the first reserpine injection.

### 2.8 Histological analysis

On days 4 and 11 following the first reserpine injection, the mice were euthanised, and their brains were promptly removed and fixed in 10 % formalin for 48 hours. Haematoxylin and eosin (H&E) staining following the standard protocol outlined by Alqurashi et al. (Alqurashi et al., 2022) was used for histopathological analysis. Briefly, after 16 hours of tissue processing using the Spin Tissue Processor STP120 (Especialidades Médicas Myr, S.L., Tarragona, Spain), the midsagittal hemisected brains were embedded in paraffin wax and sectioned into 4-µm-thick slices using a microtome (LEICA RM 2255, Leica Microsystems, Germany). The slides were stained with H&E using the Myr AutoStainer (Especialidades Médicas Myr, S.L.) and examined under a BX53 light microscope (Olympus Corporation, Tokyo, Japan) to assess alterations in the hippocampus and thalamus. Representative areas were captured at 100×, 200×, and 400× magnification using an Olympus DP73 camera, and the images were processed using Olympus CellSens Entry software.

### 2.9 Neurotransmitter analysis

For biochemical estimations, the mice were sacrificed using the cervical dislocation method. The brains and spinal cords were carefully isolated to avoid any damage and stored at −80°C. On the processing day, the minced brain and spinal cord samples were weighed and added to a homogenisation buffer (1 ml for every 0.1 g of tissue). The tissues were thoroughly homogenised and then centrifuged for 10 minutes at 1000 *g* and 4°C. The resulting supernatant was collected to estimate neurotransmitters, including serotonin, NE, and glutamic acid, and the cytokines interleukin (IL)-1β and tumour necrosis factor (TNF)-α.

#### 2.9.1 Measurement of serotonin and NE contents

Serotonin and NE levels were determined using enzyme-linked immunosorbent assay (ELISA) kits (SEKSM-0016 and SEKSM-0019, respectively, Beijing Solarbio Science & Technology Co., Ltd., Beijing, China), following the manufacturer’s instructions. Briefly, the test involved adding 50 µl of samples and standards to each well of a microplate, followed immediately by 50 µl of a working solution of biotin-conjugated anti-ST/5-HT antibody. The plate was incubated for 45 minutes at 37°C. After washing the plate, 100 µl of streptavidin-labelled detection antibody was added, and the plate was incubated again for 30 minutes at 37°C. After aspirating and washing three times, 90 µl of substrate solution was added, followed 30 minutes later by 50 µl of stop solution. Immediately after adding this solution, the optical absorbance of each well was read at 450 nm using a microplate reader (BioTek Instruments, Inc., Winooski, VT, USA). The serotonin and NE contents were determined by comparing the absorbance values with the calibration plot for standard solutions.

#### 2.9.2 Measurement of the glutamic acid content

The glutamate levels were assessed using the Glutamic Acid Content Assay kit (WST-1 chromogenic method, Beijing Solarbio Science & Technology Co., Ltd.). The absorbance was measured at 450 nm using a microplate reader (BioTek Instruments, Inc.). The glutamic acid content was determined by comparing the absorbance values with the calibration plot for standard solutions.

#### 2.9.3 Measurement of cytokine levels

The IL-1β and TNF-α levels were determined using ELISA kits (SEKM-0002 and SEKM-0034, respectively, Beijing Solarbio Science & Technology Co., Ltd.). Briefly, the procedure consisted of adding 100 µl of the samples and standards to microplate wells and incubating them at 37°C for 90 minutes. After washing the plate, 100 µl of a working solution of biotin-conjugated anti-mouse IL-1β or TNF-α antibody was added to each well. The plate was incubated at 37°C for 60 minutes. After washing the plate again, 100 µl of the streptavidin-labelled detection antibody was added to the microplate wells, and the plate was incubated at 37°C for 30 minutes. After aspirating and washing three times, 100 µl of substrate solution was added. Fifteen minutes later, 50 µl of stop solution was added, and the optical absorbance of each well was read at 450 nm using a microplate reader (BioTek Instruments, Inc.). The IL-1β and TNF-α levels were determined by comparing the absorbance values with the calibration plot for standard solutions.

### 2.10 Statistical analysis

The data were analysed and plotted using GraphPad Prism 10 software for Windows (GraphPad Inc., Sand Diego, CA, USA). The results are presented as the mean ± standard error of the mean. For the time-course measurements, data were collected from the same mice at each time point. Normality was assessed using the Shapiro-Wilk test. The data were analysed using one-way or two-way analysis of variance (ANOVA), followed by Tukey’s post hoc test for multiple comparisons. Statistical significance was defined as P < 0.05.

## 3 Results

### 3.1 Reserpine significantly and dose-dependently affected the body weight of mice

**Fig. 2:**
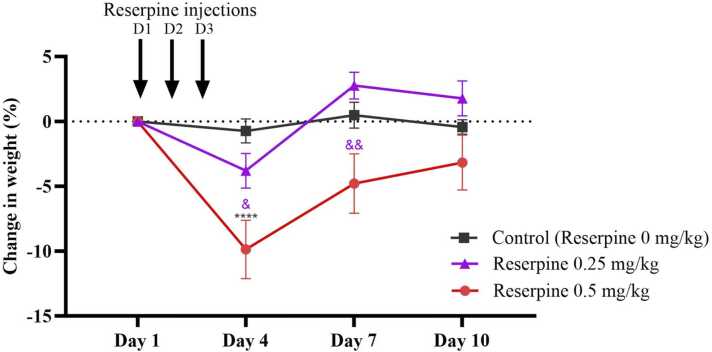
The percentage of weight change in SWR mice injected subcutaneously with vehicle or reserpine (0.25 mg/kg or 0.5 mg/kg) for 3 consecutive days. Each point represents the mean, and the vertical lines indicate the SEM for n =11–13. ****p < 0.0001 vs. control group. ^&^p < 0.05 and ^&,&^p < 0.01 vs. 0.25 mg/kg reserpine group. Two-way repeated-measures ANOVA was used, followed by Tukey’s post-hoc test.

The mice dosed at 0.5 mg/kg manifested a significant decrease in body weight, while those dosed at 0.25 mg/kg presented a moderate and short-term decrease in body weight (Fig. 2). Two-way repeated-measures ANOVA revealed that the reserpine dose had a significant effect on body weight (*F* [6139] = 3.003, *P* = 0.0086). *Post hoc* analysis with Tukey’s test showed that on day 4 (after three consecutive days of reserpine administration), the body weight had decreased significantly in the 0.5 RES group (-9.875 % ± 2.244 %) compared with the control group (-0.737 % ± 0.921 %; P < 0.0001) and the 0.25 RES group (-3.800 % ± 1.337 %; P = 0.0214). The significant difference between the 0.5 and 0.25 RES groups continued up to day 7 (P = 0.0011). Interestingly, after their drop in weight on day 4, the 0.25 RES group regained more weight than the control group on day 7 (2.762 % ± 1.033 % vs. 0.481 % ± 0.990 %) and day 10 (1.767 % ± 1.345 % vs. –0.430 % ± 0.551 %). However, there were no significant differences between the 0.5 RES and the control groups on days 7 and 10. It is important to note that despite a significant decrease in body weight in mice, neither reserpine dose caused death.

### 3.2 Reserpine with both doses induced mechanical allodynia and evoked thermal nociception in mice

**Fig. 3:**
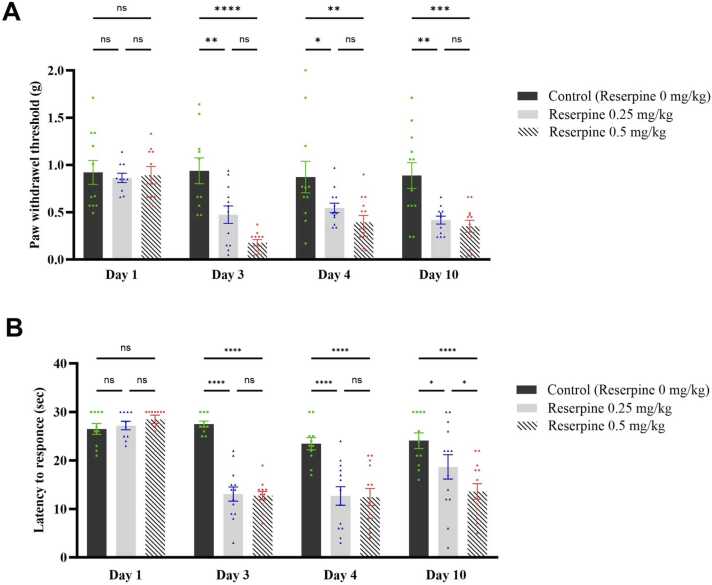
Effect of both doses of reserpine on mechanical hypersensitivity and thermal hyperalgesia tested at baseline and post-reserpine injections. (A) Paw withdrawal threshold in the von Frey test, (B) Latency to response to hot stimulus in the hot plate test. Each bar represents the mean, and the vertical lines indicate the SEM of 11–13 mice/group. *p < 0.05, **p < 0.01, ***p < 0.001, and ****p < 0.0001. Two-way repeated-measures ANOVA was used, followed by Tukey’s post-hoc test.

We conducted a series of tests to evaluate mechanical and thermal hyperalgesia in mice to assess the impact of reserpine on pain and sensitivity. First, we assessed the mechanical threshold using manual von Frey filaments at baseline and on days 3, 4, and 10 (Fig. 3**A**). Two-way repeated-measures ANOVA revealed a significant reserpine dose effect on the paw withdrawal threshold (F [6125] = 2.538, P = 0.0236). *Post hoc* analysis with Tukey’s test indicated that the paw withdrawal threshold decreased significantly on days 3, 4, and 10 following both doses of reserpine. Interestingly, the change in the 0.5 RES group was greater than the change in the 0.25 RES group on all days compared with the control group (Fig. 3**A**).

We investigated whether the two reserpine doses had a differential effect on the processing of thermal nociception by using the hot plate test. Two-way repeated-measures ANOVA showed a significant time × reserpine dose interaction on the thermal hyperalgesia in mice (*F* [6127] = 6.406, *P* < 0.0001). *Post hoc* analysis with Tukey’s test showed a significant reduction in the latency to respond to heat stimulus on day 3 and day 4 in the 0.25 and 0.5 RES groups compared with the control group (P < 0.0001; Fig. 3**B**). The reduction in the thermal hyperalgesia lasted until day 10 in the 0.5 RES group (P < 0.0001) and the 0.25 RES group (P = 0.0394), although by that time, the effect was more pronounced in the 0.5 RES group.

**Fig. 4:**
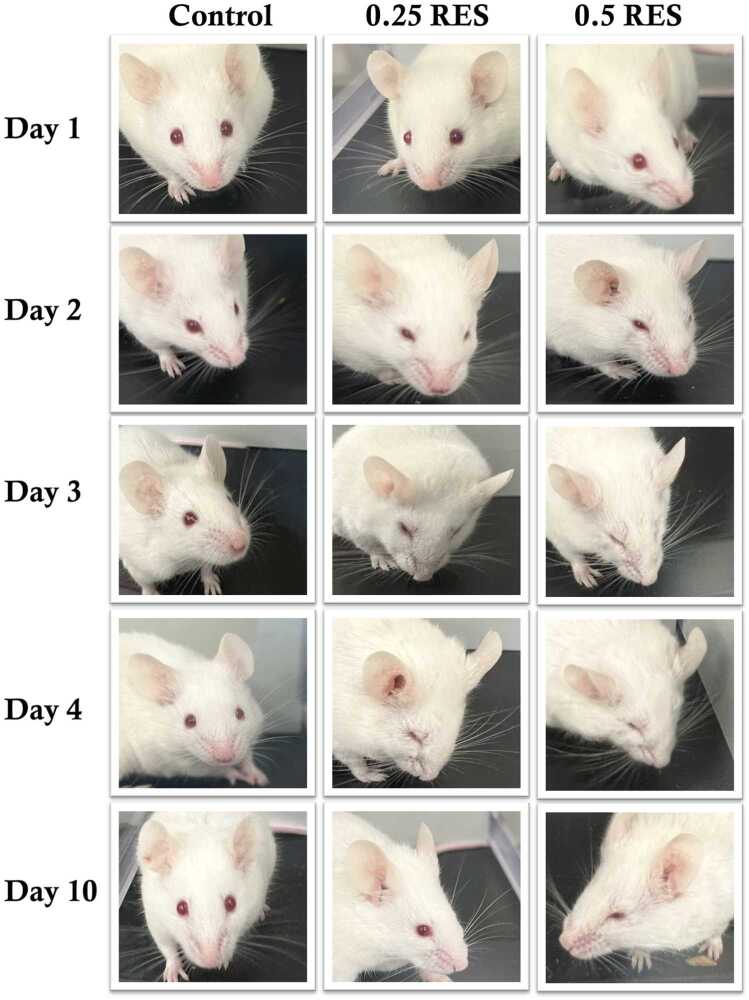
Effect of both reserpine doses on qualitative pain assessment based on Grimes scale parameters in mice. The facial expression of randomly selected mice from each group at baseline (day 1), then following each dose of reserpine or vehicle by 24 hours (days 2, 3, and 4), and lastly at the end of the study (day 10). Pictures showed different parameters used to assess the Grimace scale (closing eye, nose and cheek bulging, ear position, and whiskers change) indicating moderate to severe pain in mice received 0.25 mg/kg reserpine (0.25 RES) and 0.5 mg/kg reserpine (0.5 RES).

Regarding the qualitative pain responses in mice based on the Grimace Scale parameters, as expected, the eyes were fully closed in all mice that received reserpine on day 4. These changes in facial expression – that is, eyelids drooped, ear position, nose and cheek plugs, and whiskers changes – remained up to day 10 in the 0.5 RES group (Fig. 4). However, the facial expressions of pain had disappeared by day 10 in the 0.25 RES group.

Taken together, these results suggest that the 0.5 mg/kg dose of reserpine elicits more robust and enduring effects on mechanical and thermal sensitivity, as well as pain-related facial expressions, compared with the 0.25 mg/kg dose.

### 3.3 Reserpine significantly reduced spontaneous and forced activities and impacted the grip strength of mice

Impaired motor balance during daily activities and an elevated risk of falling are key symptoms of FM (Chiaramonte, Bonfiglio, & Chisari, 2019). Therefore, it is crucial to evaluate the impact of different doses of reserpine on locomotor activity in mice. We assessed spontaneous motor behaviour in the form of the total distance moved and velocity using the open-field test. Reserpine significantly reduced the total distance moved (*F* [4,63] = 3.917, P = 0.0067) and velocity (*F* [4,62] = 2.758, P=0.0355).

**Fig. 5:**
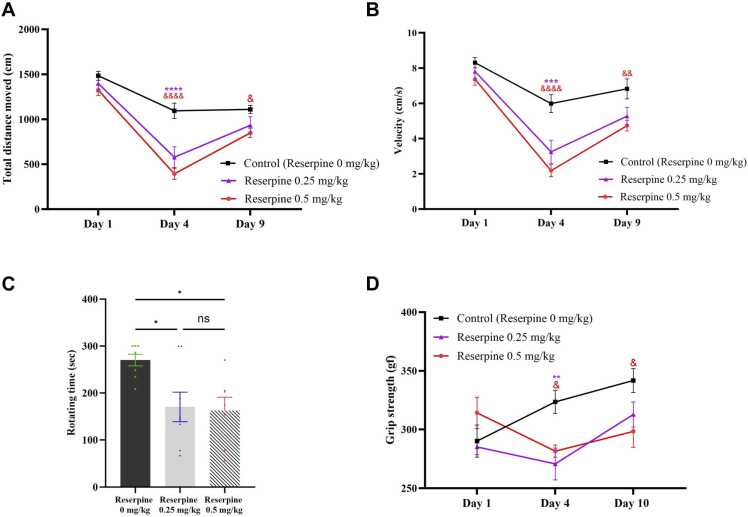
Effect of both doses of reserpine on motor activities and muscle strength in mice tested at baseline and post-reserpine injections. Reserpine injections with 0.5 mg/kg dose for three consecutive days reduced all motor outcomes in different time point measurements compared to the control group. (A) TDM, (B) velocity, (C) rotating time in the rotarod test, and (D) Grip strength. In line graphs, each point represents the mean, and the vertical lines indicate the SEM for n =7–8. **P<0.01, ***P < 0.001, and ****p < 0.0001 vs. 0.25 mg/kg reserpine group. ^&^p < 0.05, ^&,&^p < 0.01, and ^&,&,&,&^p ^<^ 0.0001 vs. 0.5 mg/kg reserpine group. In the C graph, each bar represents the mean, and the vertical lines indicate the SEM for 7–8 mice/group where *p < 0.05. One-way or Two-way repeated-measures ANOVA was used, followed by Tukey’s post-hoc test.

*Post hoc* analysis with Tukey’s test revealed that the total distance moved was significantly lower in the 0.5 RES group compared with the control group on day 4 (P < 0.0001) and day 9 (P = 0.0496). The total distance moved also decreased significantly in the 0.25 RES group on day 4 compared with the control group (P < 0.0001), but there was no difference between the groups on day 9 (Fig. 5**A**).

Similarly*,* the 0.5 RES group presented a marked decrease in velocity on day 4 (P < 0.0001) and day 9 (P = 0.0077) compared with the control group. Velocity was decreased significantly in the 0.25 RES group on day 4 compared with the control group (P=0.0002), but there was no difference between the groups on day 9 (Fig. 5**B**).

Based on these data, the 0.5 mg/kg reserpine leads to more pronounced effects on spontaneous motor activities compared with the 0.25 mg/kg dose on day 4. Moreover, the higher dose is more effective in sustaining motor deficits until day 6 post-induction.

Two-way repeated-measured ANOVA also showed that reserpine dose significantly affect forced motor activity in the rotarod test (*F* [2,20] = 5.697, P = 0.0110). *Post hoc* analysis with Tukey’s test revealed significant difference between the 0.25 and 0.5 RES groups compared with the control group (P = 0.0257 and P = 0.0202, respectively; Fig. 5**C**).

The alterations in spontaneous and forced activities following reserpine injections were accompanied by a significant reduction in grip strength (two-way repeated-measures ANOVA: *F* [4,62] = 3.357, P = 0.0150). Based on *post hoc* analysis with Tukey’s test, on day 4 grip strength was significantly lower in the 0.5 RES (P = 0.0341) and 0.25 RES (P = 0.0041) groups compared with the control group. On day 10, only the 0.5 RES group presented a significant reduction in grip strength compared with the control group (P = 0.0216; Fig. 5**D**).

### 3.4 Both doses of reserpine induced depressive-like behaviour in mice

Next, we investigated whether the tested reserpine doses evoked comorbidities associated mostly with FM. Specifically, we considered the grooming time in the splash test and the immobility time in the forced swimming and tail suspension tests.

**Fig. 6:**
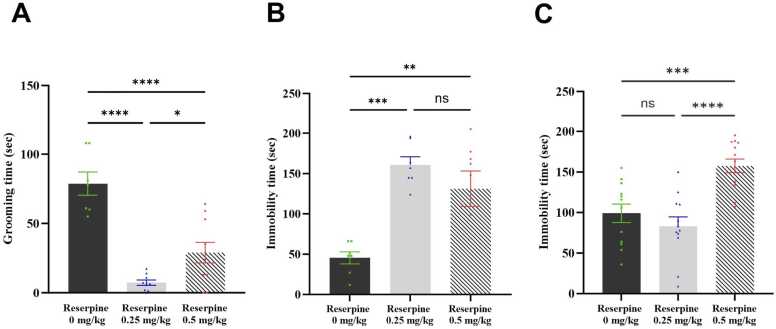
Effect of both doses of reserpine on depressive-like behaviour in mice. (A) Grooming time in the Splash test, (B) Immobility time in the Forced swimming test, and (C) Immobility time in the Tail suspension test. Each bar represents the mean, and the vertical lines indicate the SEM of 7–10 mice/group in ST and FST tests; and 12–13 mice/group in TST. *p < 0.05, **p < 0.01, ***p < 0.001, and ****p < 0.0001. One-way ANOVA was used, followed by Tukey’s post-hoc test.

Regarding the splash test, one-way ANOVA showed a significant reserpine dose effect on the duration of grooming after spraying 10 % sucrose on the dorsal coat (*F* [2,23] = 29.02, P < 0.0001). *Post hoc* analysis with Tukey’s test revealed that both doses of reserpine decreased the grooming time significantly compared with the control (P < 0.0001; Fig. 6**A**).

For the forced swimming test, one-way ANOVA showed a significant reserpine dose effect on the duration of immobility (*F* [2,19] = 14.18, P=0.0002). *Post hoc* analysis with Tukey’s test revealed that the immobility time was significantly lower in the 0.25 and 0.5 RES groups compared with the control group (P = 0.0002 and P = 0.0025, respectively; Fig. 6**B)**.

Lastly, one-way ANOVA demonstrated a significant reserpine dose effect on the duration of immobility in the tail suspension test (*F* [2,34] = 14.59, P<0.0001). The 0.5 RES group showed a significantly increased immobility time compared with the control group (P = 0.0010). However, there was no significant difference in immobility time between the 0.25 RES group and the control group (Fig. 6**C**).

We also assessed the mice's anxiety-like behavior and memory using central preference, elevated pulse maze, and Y-maze tests. For additional information, please refer to Supplementary File 1.

### 3.5 Histopathological analysis of pain and cognition-related structures in mice following reserpine administration

**Fig. 7:**
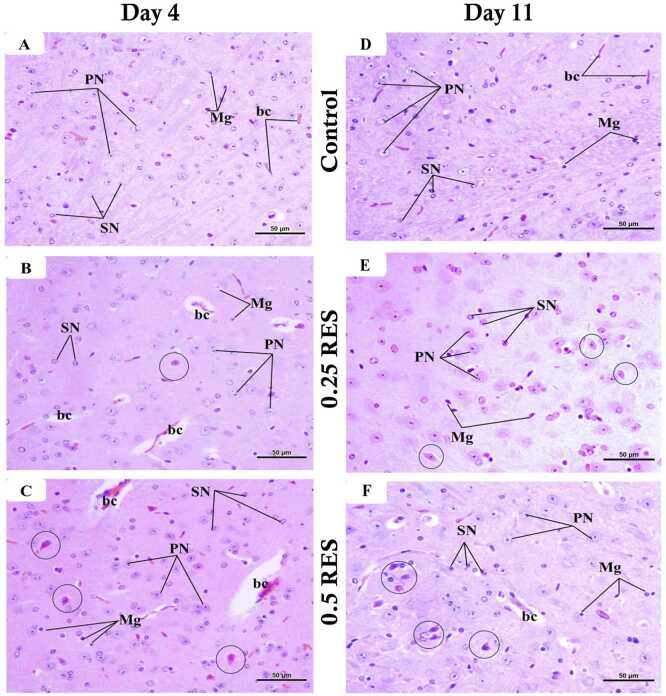
Representative photomicrographs of H&E-stained thalamus sections. (A, D) The control group exhibited a normal thalamic structure, with large principal cells (PN) and small neurons (SN). Numerous microglial cells (Mg) and tiny capillaries (bc) were also present. (B, E) The 0.25 mg/kg reserpine (0.25 RES) group showed a structure similar to the control group but with some degenerated PN marked by black circles, which were more abundant on day 11. (C, F) The 0.5 mg/kg reserpine (0.5 RES) group displayed many degenerated PN indicated by black circles and the presence of dilated bc. The scale bar corresponds to 50 µm (H&E x 400).

We used histology to examine changes in pain and cognition-related structures, specifically the thalamus and hippocampus, to identify changes associated with reserpine administration. Fig. 7 presents H&E-stained sagittal sections of the thalamus from the three groups. In the control group, the thalamus exhibited normal histology at both time points, with an abundance of healthy neurons on a pinkish background (neuropil). The primary cell type was large principal cells with pale, rounded nuclei, alongside smaller cells with dark nuclei, microglia with small dark nuclei, nerve fibres, and tiny capillaries.

On day 4, the thalamic structure in the 0.25 RES group appeared similar to that of the control group, although there were some degenerated principal cells and slightly dilated capillaries. In the 0.5 RES group, more principal cells appeared condensed with dark cytoplasm, and there were more dilated capillaries. By day 11, the thalamic structure of the 0.25 RES group still resembled that of the control group, but with some degenerated principal cells. In the 0.5 RES group, there was a noticeable increase in degenerated and condensed principal and small cells, as well as more dilated capillaries. Unexpectedly, there were more histological changes in the 0.25 and 0.5 RES groups on day 11 than on day 4, suggesting that the effects of reserpine on the thalamus worsens over time.

**Fig. 8:**
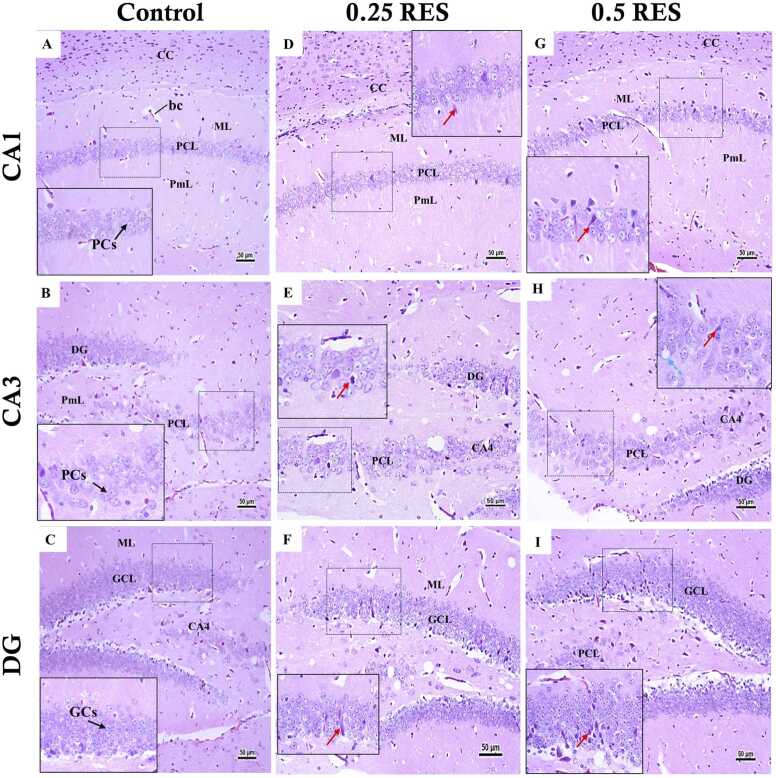
The changes in the hippocampus on day 4 post first reserpine injection. The images show the characterization of three distinct areas of the hippocampus, CA1, CA3, and dentate gyrus (DG) from the control group, the 0.25 mg/kg reserpine group (0.25 RES), and the 0.5 mg/kg reserpine group (0.5 RES): (A, B) CA1 and CA3 of the control group showed normal pyramidal cells, and (C) The control’s DG also observed normal granular cells. (D) CA1 in 0.25 RES has shrunken dark degenerated pyramidal cells (red arrow). (E) The CA3 in 0.25-reserpine has degenerated pyramidal cells (red arrow). (F) The DG of the 0.25 RES group appeared with degenerated granular cells (red arrow). In all areas of the 0.5 RES group (G, H, I), the same finding of the 0.25 RES group was detected but with more dominance, and obvious changes. Scale bar corresponds to 50 µm (H&E x 200 or 400). CC: Corpus Callosum, ML: molecular layer, PCs: Pyramidal cells. PCL: Pyramidal Cell Layer, PmL: Polymorphic layer, GCL: Granular cell layer, GCs: Granular cells.

**Fig. 9:**
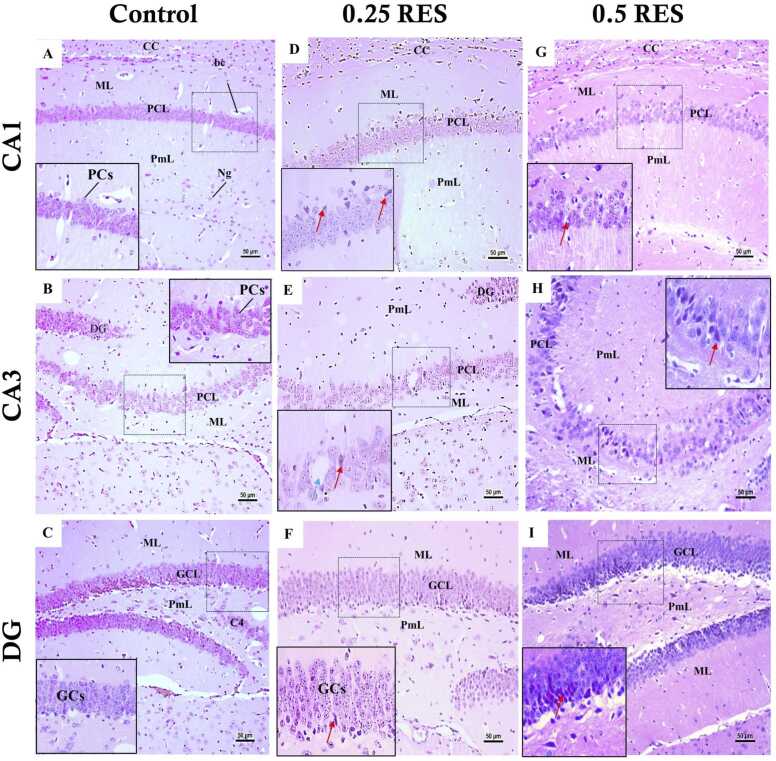
The changes in the hippocampus on day 11 post first reserpine injection. The figure shows the characterization of three distinct areas of the hippocampus, CA1, CA3, and dentate gyrus (DG) from the control group, the 0.25 mg/kg reserpine group (0.25 RES), and the 0.5 mg/kg reserpine group (0.5 RES): (A, B) The control group's CA1 and CA3 regions display normal, large, pale vesicular nucleated multilayer pyramidal cells. (C) The DG area in the control group shows normal morphology with healthy granular cells (GCs). (D) In the 0.25 mg/kg reserpine group, the CA1 region exhibits shrunken, dark, degenerated pyramidal cells (red arrow). (E) The CA3 region in the 0.25 mg/kg reserpine group shows degenerated pyramidal cells (red arrow) and tissue vacuolation (light blue arrow). (F) The DG of the 0.25 mg/kg reserpine group contains one layer of degenerated neurons. (G) In the 0.5 mg/kg reserpine group, similar findings to the 0.25 mg/kg reserpine group are detected in the CA1 region but with a higher number of degenerated neurons. (H) the CA3 region of the 0.5 RES group appeared with a higher number of degenerated neurons (red arrow) compared to the 0.25 mg/kg reserpine group. (I) In the DG of the 0.5 RES group, the degenerated neurons (red arrow) almost reach the full thickness of the granular cell layer. The scale bar corresponds to 50 µm (H&E x 200 or 400). bc: blood capillaries, CC: Corpus Callosum, CGL: granular cell layer, GCs: Granular cells, ML: molecular layer, PCs: Pyramidal cells. PCL: Pyramidal Cell Layer, PmL: Polymorphic layer, Ng: neuroglial cells.

In the hippocampus, the control group on day 4 (Fig. 8) and day 11 (Fig. 9) displayed normal histology. The CA1 and CA3 regions had healthy pyramidal cells characterised by pale, basophilic nuclei with visible chromatin material. Similarly, the dentate gyrus exhibited normal granular cells. In contrast, on day 4 (Fig. 8) the 0.25 and 0.5 RES groups presented degenerated, shrunken, and dark pyramidal cells in the CA1 and CA3 regions. The CA3 region also exhibited vacuolation in the polymorphic and pyramidal cell layers. The dentate gyrus in the 0.25 and 0.5 RES groups showed degenerated granular cells, with the latter group displaying more extensive degeneration across multiple cell layers within the dentate gyrus. By day 11 (Fig. 9), the reserpine-treated groups exhibited more pronounced histopathological alterations in the hippocampus. The pyramidal cell layers of the CA1 and CA3 regions appeared disorganised, with numerous cells showing degenerative changes such as shrunken cell bodies and dark cytoplasm. The molecular and polymorphic layers had increased neuroglial cells, vacuoles, and dilated blood capillaries. In the dentate gyrus, the granular cell layer contained many degenerated and shrunken granular cells with condensed nuclei, especially in the 0.5 RES group, where degeneration extended through almost the entire thickness of the dentate gyrus.

### 3.6 The 0.5mg/kg reserpine dose induced greater neurotransmitter dysregulation than the 0.25mg/kg dose

**Fig. 10:**
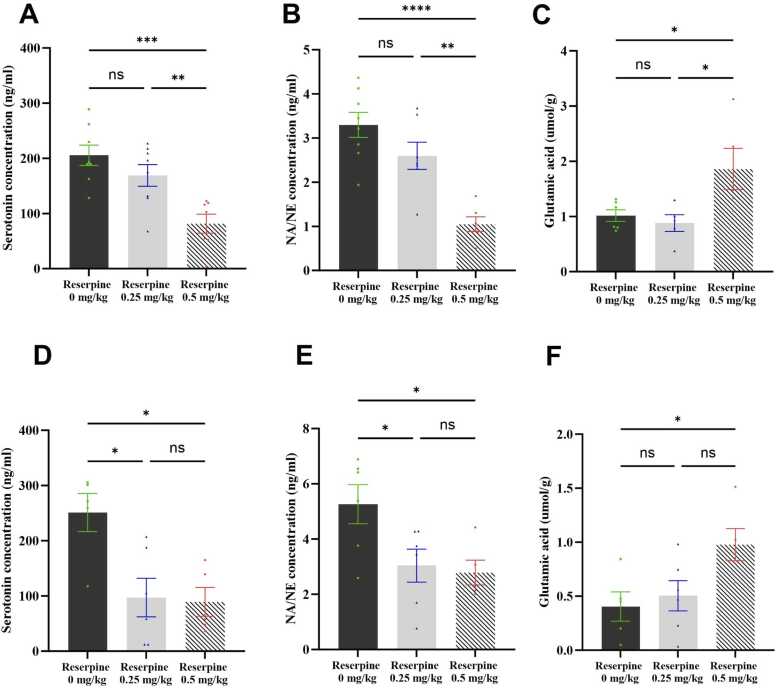
Effect of reserpine on neurotransmitter levels in the brain and spinal cord. (A) Serotonin level in the brain, (B) Norepinephrine level in the brain, (C) Glutamate level in the brain, (D) Serotonin level in the spinal cord, (E) Norepinephrine level in the spinal cord, and (F) Glutamate level in the spinal cord. Each bar represents the mean, and the vertical lines indicate the SEM of 6–8 mice/group. *p < 0.05, **p < 0.01, ***p < 0.001, and ****p < 0.0001. One-way ANOVA was used, followed by Tukey’s post-hoc test.

Fig. 10 shows the effect of reserpine on the brain and spinal cord serotonin, NE, and glutamate levels. One-way ANOVA revealed a significant reserpine effect on serotonin levels (F [2,20] = 11.00, P = 0.00005), NE levels (F [2,9] = 37.51, P < 0.0001), and glutamate levels (F [2,13] = 5.264, P = 0.0211) in the brain. *Post hoc* analysis with Tukey’s test revealed that the serotonin and NE levels decreased significantly in the 0.5 RES group compared with the control group (P = 0.0004 and P < 0.0001, respectively). There were no significant changes between the 0.25 RES and control groups. The glutamate level increased significantly only in the 0.5 RES group compared with the control group (P = 0.0473)

In the spinal cord, one-way ANOVA revealed a significant reserpine effect on the serotonin (*F* [2,13] = 7.544, P = 0.0067), NE (*F* [2,14] = 5.045, P = 0.0224), and glutamate (F [2,13] = 4.424, P = 0.0342) levels. *Post hoc* analysis with Tukey’s test revealed that the serotonin and NE levels decreased significantly in the 0.5 RES group compared with the control group (P = 0.0123 and P = 0.0351, respectively. Similarly, the serotonin and NE levels decreased significantly in the 0.25 RES group compared with the control group (P = 0.0128 and P = 0.0474, respectively). The glutamate levels increased significantly in the 0.5 RES group compared with the control group (P = 0.0401), but there was no difference between the 0.25 RES and control groups (Fig. 10**F**).

In summary, while the 0.25 mg/kg dose of reserpine only affected the serotonin and NE levels in the spinal cord, the 0.5 mg/kg dose induced significant changes in the serotonin, NE, and glutamate levels in both the brain and spinal cord, highlighting the more pronounced effect of the higher reserpine dose.

### 3.7 Both doses of reserpine affected proinflammatory cytokine levels

**Fig. 11:**
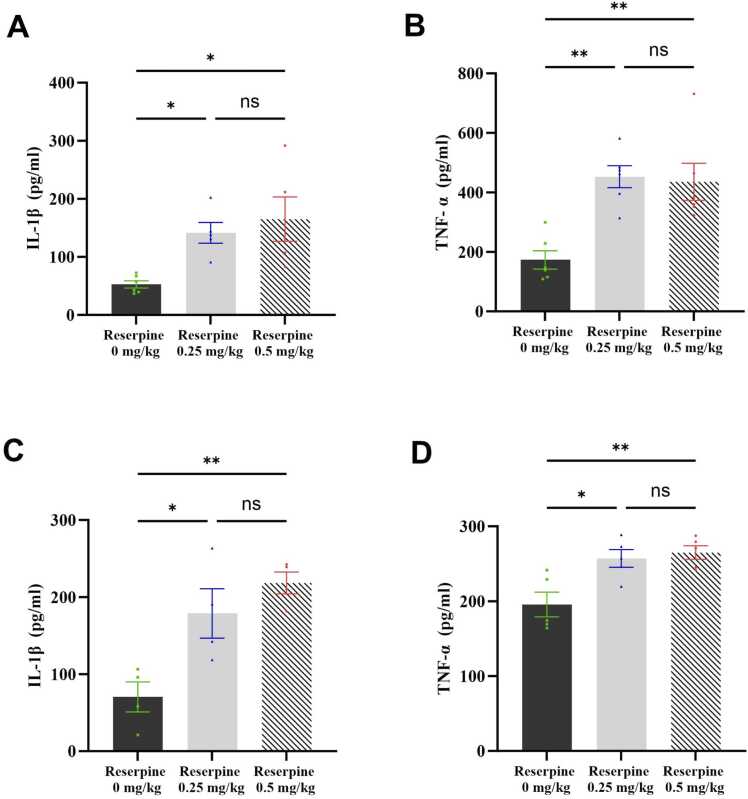
Effect of both doses of reserpine on proinflammatory cytokine levels in the brain and spinal cord. (A) IL-1β levels in the brain, (B) TNF-α levels in the brain, (C) IL-1β levels in the spinal cord, and (D) TNF-α levels in the spinal cord. Each bar represents the mean, and the vertical lines indicate the SEM of 4–6 mice/group. *p < 0.05, **p < 0.01, ***p < 0.001, and ****p < 0.0001. One-way ANOVA was used, followed by Tukey’s post-hoc test.

Fig. 11**A-B** shows the effect of reserpine on brain IL-1β and TNF-α levels. One-way ANOVA revealed a significant reserpine effect on the IL-1β (*F* [2,13] = 6.927, P = 0.0090) and TNF-α (*F* [2,15] = 11.94, P = 0.0008) levels in the brain. Based on *post hoc* analysis with Tukey’s test, the IL-1β and TNF-α levels increased significantly in the 0.5 RES group compared with the control group (P = 0.0105 and P = 0.0026, respectively). Similarly, the IL-1β and TNF-α levels increased significantly in the 0.25 RES group compared with the control group (P = 0.0409 and P = 0.0015, respectively).

There were similar patterns in the spinal cord. One-way ANOVA revealed a significant reserpine effect on the IL-1β (*F* [2,9] = 10.97, P = 0.0039) and TNF-α (*F* [2,12] = 8.830, P = 0.0044) levels. *Post hoc* analysis with Tukey’s test revealed that the IL-1β and TNF-α levels increased significantly in the 0.5 RES group compared with the control group (P = 0.0037 and P = 0.0062, respectively), and in the 0.25 RES group compared with the control group (P = 0.0221 and P = 0.0135, respectively).

## 4 Discussion

Research designed to select and optimise the dose of a specific agent to induce a model of a disease in rodents is crucial for ensuring the reliability and reproducibility of the experimental results. Moreover, an optimised dosing regimen helps to accurately mimic the disease state, thereby providing a robust platform to study pathophysiology and to evaluate potential therapeutic interventions (McGonigle & Ruggeri, 2014). We delved into this strategy by investigating the impact of different doses of reserpine to provoke FM in mice. In studies using reserpine to induce FM-like symptoms in mice, the commonly used doses are 0.25 mg/kg (de Souza et al., 2014, Klein et al., 2014, Peres Klein et al., 2016), 0.5 mg/kg (Singh, Kaur, Singh, et al., 2021), or 1 mg/kg (Brusco et al., 2021; Fischer et al., 2020; Xu et al., 2013). These doses are typically administered subcutaneously once a day for three consecutive days. However, the reasons for the variations in reserpine doses among the published articles are often not explicitly clarified. Additionally, variations in the effects of reserpine on behavioural tasks in mice across studies have not been discussed (Brum et al., 2020; Sałat & Furgała-Wojas, 2021; Singh et al., 2020). Hence, we compared the impacts of common reserpine doses in inducing FM in male SWR mice, specifically 0.25 and 0.5 mg/kg. We employed a comprehensive array of behavioural assessments alongside pertinent histopathological examination of the brain to pinpoint the most effective dosage for eliciting FM symptoms in mice. By clarifying the optimal dose of reserpine to induce FM, we can effectively contextualise the findings and their implications for understanding and treating FM.

Neurotransmitter alterations, such as decreased serotonin and NE levels and increased glutamate levels, are considered one of the primary pathologies of FM (Becker & Schweinhardt, 2012). Therefore, reserpine is particularly valuable in FM research because of its ability to decrease serotonin and NE levels, through its action as a competitive agonist at the VMAT-2 receptor, preventing the release of biogenic amines in the brain (Brum, Becker, Fialho, & Oliveira, 2022; Yaffe et al., 2018). Disruption of serotonin and NE signalling triggers inflammatory processes and increases IL-1β and TNF-α levels in the central nervous system (Baganz and Blakely, 2013, O'Donnell et al., 2012), therefore leading to increased glutamate production (Ye et al., 2013), closely mirroring the neurochemical imbalance observed in patients with FM.

Given these neurochemical alterations, it is important to consider the broader physiological impacts, including changes in body weight after reserpine administration to experimental animals. Our results indicate that both reserpine doses decreased the body weight of the treated mice, with recovery of body weight after stopping the reserpine injections. However, the body weight reduction of mice that received the 0.25 mg/kg dose of reserpine was mild and they had a faster regain of weight than the control group after only two days of reserpine withdrawal. Based on prior research, a reduction in body weight that is accompanied by fast recovery is mostly due to decreased food intake (Park et al., 2018). Serotonin and NE depletion can contribute to changes in feeding behaviour and alter metabolism (Leibowitz and Alexander, 1998, Wellman, 2000), which may lead to weight loss in rodents following reserpine administration. Furthermore, the effect of reserpine on body weight in mice is possibly due to its impact on pain perception and motor performance, which contribute to reduce food intake. Additionally, our results demonstrated that while reserpine exposure initially reduced body weight, the weight gain of the 0.25 RES group exceeded that of the control group once the reserpine injections ended. Moreover, mice in the 0.5 RES group regained weight more rapidly compared with the other groups relative to their initial weight loss. This phenomenon could be explained by the mice overeating in an attempt to compensate for the energy deficiency experienced during reserpine treatment (Harris et al., 1998). In line with our findings, several studies have reported similar effects of reserpine at doses of 0.25 and 0.5 mg/kg on the weight of various mouse strains, including ICR, C57BL/6 J, and Swiss albino mice (Kaur et al., 2019, Nagakura et al., 2018, Park et al., 2018). Conversely, some studies using the same mouse strain and a similar age and weight as we used in the present study reported that reserpine did not affect body weight (Brusco et al., 2019; Fischer et al., 2020). The discrepancies observed across the available studies suggest that other factors beyond the pharmacodynamic effects of reserpine affect body weight. These factors may include variations in laboratory conditions, dietary intake, handling procedures for mice, and the specific site chosen for subcutaneous injection.

Mechanical allodynia and thermal hyperalgesia are prominent features of FM (Maugars, Berthelot, Le Goff, & Darrieutort-Laffite, 2021). In the present study, we observed the development of mechanical and thermal allodynia in mice treated with both doses of reserpine, consistent with previous studies using doses of 0.25 mg/kg (Sałat & Furgała-Wojas, 2021), 0.5 mg/kg (Singh, Kaur, Singh, et al., 2021), and 1 mg/kg (Brum et al., 2020) in mice. According to Nagakura et al., sensory hypersensitivity in rodents after repeated reserpine injections results from a reduction in biogenic amines in the nervous system (Nagakura et al., 2009). We aimed to assess the enduring impact of various reserpine doses up to 10 days after the initial injection. As predicted, the significant effects persisted until day 10 for both reserpine doses, consistent with previous findings. In previous studies, the impact of a 0.25 mg/kg reserpine dose on the von Frey and tail-flick tests remained until day 14 after the initial injection (Ferrarini et al., 2022, Martins et al., 2022). Notably, we revealed that the persistent effect of the 0.5 mg/kg reserpine dose on facial expression of pain, as measured by the Grimace Scale, remained evident until day 10, while it disappeared in the 0.25 RES group. However, other research indicates that the prolonged effect of reserpine on the Grimace Scale can extend for up to 28 days in female mice (Ferrarini et al., 2021), and up to 17 days in male rats (Tanei, Miwa, Yoshida, Miura, & Nagakura, 2020). These variations in findings may be attributed to differences in rodent species and sex.

While FM primarily manifests as symptoms related to pain, some patients may also experience motor defects or impairments. One of the prevalent associated symptoms of FM is fatigue, which affects about 76 % of patients with FM (Vincent et al., 2013). Moreover, fine and gross motor control tests have revealed several abnormalities that affect patients with FM (Auvinet et al., 2011, Canny et al., 2009, Pérez-de-Heredia-Torres et al., 2013). Previous studies have reported that three consecutive injections of reserpine in laboratory animals at various doses are associated with several motor defects. These include a reduced number of rearing and square crossings (Klein et al., 2014, Singh et al., 2020), deterioration of muscle coordination (Brum et al., 2020; Yao, Li, Kandhare, Mukherjee-Kandhare, & Bodhankar, 2020), and decreased muscle strength (Fischer et al., 2020). In the present study, we used various motor assessment techniques to explore potential differences in motor performance, fatigue, and muscle strength induced by the two tested reserpine doses. We found that both doses significantly impacted motor performance in mice. However, it is essential to highlight the diverse findings reported in the literature regarding the effects of reserpine on motor behaviour. For example, certain studies have emphasised the acute influence of reserpine, at a dose of 0.5 mg/kg, across all tested motor behaviours (Singh et al., 2020). Conversely, there have been contrasting results in other research, where the administration of a 1 mg/kg dose to male Swiss mice showed no discernible effect on motor performance (Brusco et al., 2019). Additionally, complexities have arose within studies themselves, with some reporting the influence of reserpine solely on specific motor tests, such as grip strength, while finding no effect on other measures such as distance moved and velocity (Brusco et al., 2021; Fischer et al., 2020). These differences among the studies regarding the effect of reserpine on motor behaviours may represent a limitation of the reserpine mouse model of FM. However, elucidating the exact reasons behind these discrepancies is challenging. Potential factors such as strain differences, age, and/or variations in experimental settings may contribute to the discrepancies among the published studies.

Mood disorders are a significant concern among the comorbid symptoms of FM (Blasco-Serra et al., 2015). For example, a bidirectional relation has been identified between FM and depression, with FM greatly increasing the risk of depression and genetic predisposition to depression slightly increasing the risk of FM (Gau, Hung, Chuang, & Wei, 2023). Repeated reserpine treatment is an attractive murine model of FM because this chemical exerts a central effect on neurotransmitter levels within the brain, therefore leading to depressive-like behaviors in experimental animals along with exaggerated pain perception (Blasco-Serra et al., 2015). We found that both doses of reserpine increased pain sensitivity, which was coupled with depression-related symptoms, as indicated by their behaviour in the forced swimming, tail suspension, and splash tests. Similarly to our results, researchers have found that reserpine administration leads to depression-like symptoms in rodents in the context of FM (Ferrarini et al., 2022, Singh et al., 2021) and the emergence of anxiety-like behaviour *per se* (Brusco et al., 2019; Fischer et al., 2020; Kaur et al., 2019). Besides, recent evidence has identified a close relationship between FM and cognitive decline, including spatial memory (Glass, 2009). However, neither of the reserpine does we tested affected spatial memory, as assessed by the Y-maze test (supplementary file). In contrast to our results, researchers have found that reserpine negatively affected memory based on the Morris water maze test (Singh et al., 2020, Singh et al., 2021). This discrepancy could be explained by the fact that reserpine led to deterioration of motor behaviour in our mice, as the Y-maze test assesses the spontaneous alternation according to the number of entrances into Y-shaped arms (Kraeuter, Guest, & Sarnyai, 2019).

The histopathological changes in the hippocampus and thalamus, which are critical for sensory and cognitive processing, could be pivotal in understanding the clinical symptoms of FM, such as chronic pain, cognitive deficits, and mood disorders. Damage to these regions may amplify pain sensitivity and disrupt emotional and cognitive functions, potentially explaining the widespread pain, memory issues, and mood disturbances in patients with FM. (Tajerian et al., 2018, Wolff et al., 2021). Here, we investigated the histological changes in the brain of mice following reserpine injection. To our knowledge, this is the first study to examine the neurotoxic effects of different doses of reserpine on the hippocampus and thalamus. After the reserpine injections, histological analysis of the hippocampus revealed several notable changes, particularly in the CA1 and CA3 regions in the form of neuronal degeneration and a reduction in the density of pyramidal cells. The observed neuronal degeneration in the hippocampus may be attributed to the depletion of monoamines such as serotonin (Bombardi et al., 2021), dopamine (Zhou, Yi, Wang, Lim, & Tan, 2023), and NE (Song et al., 2019). In addition, acute excessive release of glutamate due to increased TNF-α and IL-1β production after reserpine administration could also lead to cell death due to excitotoxicity (Komoltsev et al., 2021, Singh et al., 2021). The structural damage we observed in the hippocampus aligns with previous studies indicating that monoaminergic deficits can result in significant neurodegeneration (Abd-Ellatief, Mohamed, & Kotb, 2018). Another critical finding was the increase in glia and dilated blood capillaries. These changes suggest an ongoing inflammatory response to neuronal injury (Burda & Sofroniew, 2014). We also noted marked histological alterations in the thalamus, a relay centre for sensory and motor signals (Sheridan & Tadi, 2019), following reserpine treatment. The most prominent changes included neuronal loss and the presence of vacuolated neurons, suggesting cytoplasmic degeneration. Again, the degeneration observed in the thalamus can be linked to its high dependency on monoaminergic input for proper functioning (Jacob & Nienborg, 2018). In particular, the depletion of serotonin may severely disrupt thalamic neurons, leading to the observed histopathological changes. Serotonin modulates thalamic activity and influences various neurophysiological processes, including sensory processing (Noseda, Borsook, & Burstein, 2017) Therefore, the loss of serotonergic input could contribute to the structural damage and functional deficits we observed in the thalamus. Moreover, similarly to the hippocampus, there was evidence of gliosis and dilated blood capillaries in the thalamus. Those results indicate an inflammatory response to neuronal injury, which can contribute to further neurodegeneration (Burda & Sofroniew, 2014).

We also observed more histological changes in both reserpine groups on day 11 than on day 4, suggesting that the effects of reserpine on the thalamus worsen over time. As we discussed, reserpine depletes stores of serotonin, NE, and dopamine. Early on, this depletion might cause functional changes without inducing significant structural damage. The brain might still be attempting to compensate for the loss of neurotransmitters. As depletion continues, the brain’s compensatory mechanisms may fail, leading to more pronounced functional impairments and structural damage. Chronic low levels of monoamines can result in oxidative stress, inflammation, and apoptosis, contributing to progressive tissue damage (Bombardi et al., 2021, Song et al., 2019, Zhou et al., 2023).

Lastly, this study offers valuable insights into the neurochemical alterations induced by both reserpine doses. Our results suggest a dose-dependent effect of reserpine on neurotransmitter levels in the mouse brain and spinal cord. The dose of 0.5 mg/kg significantly altered all measured parameters. In particular, it decreased serotonin and NE levels and increased glutamate levels in the brain and spinal cord, changes that are consistent with the existing literature (Kaur et al., 2019, Singh et al., 2021, Singh et al., 2021). However, the 0.25 mg/kg dose showed a more selective effect: it did not alter neurotransmitter levels in the brain but induced spinal cord changes, indicating a region-specific response. In agreement with our results, Klein et al. (Klein et al., 2014) observed similar findings with a dose of 0.25 mg/kg, emphasising the dose-dependent and site-specific effects of reserpine. One possible explanation is the difference in blood-brain barrier (BBB) permeability. The BBB is more selective in the brain, which might limit the amount of reserpine that reaches this organ when administered at a lower dose, while the spinal cord has a relatively higher permeability, allowing more of the drug to penetrate the tissue and exert its effects (Bartanusz, Jezova, Alajajian, & Digicaylioglu, 2011). Another factor to consider is the distribution of drug-metabolising enzymes in different tissues, which could also impact the effectiveness of reserpine in the brain versus the spinal cord (Zhang et al., 2022). However, both reserpine doses increased IL-1β and TNF-α levels in the brain and spinal cord, underscoring their ability to elevate proinflammatory cytokine production, even at lower concentrations.

*Study Strength* The differential effects observed between the 0.25 and 0.5 mg/kg doses of reserpine can be attributed to their distinct impacts on neurotransmitter systems and underlying cellular mechanisms. The 0.5 mg/kg dose induced more profound and sustained alterations in serotonin, NE, and glutamate levels, leading to more severe FM-like symptoms and longer-lasting effects, which persisted until day 10 after the initial reserpine dose. In contrast, the 0.25 mg/kg dose produced milder changes with a shorter duration of action. This disparity suggests that the higher dose not only triggers more intense neurochemical disruptions, but also results in prolonged neuroinflammation and increased symptom severity. The use of these doses in experimental models allows for a comprehensive evaluation of the acute and chronic aspects of FM, enhancing the relevance of the findings for developing targeted therapies and understanding the progression of FM.

*Study Limitations* While our study provides a comprehensive assessment approach, including behavioral, neurochemical, and histological evaluations, and enhances our understanding of the effects of different doses of reserpine on FM-like symptoms in mice, we must acknowledge several limitations. First, the use of a single mouse strain and sex may limit the generalisability of our findings, as different strains and sexes could exhibit varied responses to reserpine. Another limitation of this study is that we did not quantify the grimace scale, which could have provided a more objective assessment of pain-related facial expressions. Additionally, we did not perform quantitative analysis of neuronal degeneration in the histological sections, which may have limited our ability to fully assess the extent of neurodegeneration in response to reserpine treatment. These factors should be considered when interpreting the results and their applicability to broader contexts. Lastly, we did not measure neurotransmitter levels on day 4 or during the middle of the study. This limitation has restricted our understanding of the dynamic changes in neurotransmitter levels over time, making it challenging to assess how these levels fluctuate in response to reserpine administration and how they correlate with the progression of FM-like symptoms.

## 5 Conclusions

This study evaluates the effects of two commonly used doses of reserpine to mimic FM pathophysiology in mice. Through detailed behavioural assessment, this study indicated that the impact of the 0.25 mg/kg dose of reserpine was comparable to that of 0.5 mg/kg, with minimal differences in long-lasting effects. Besides, neurotransmitter analysis revealed that the 0.5 mg/kg dose induced significant changes in serotonin, NE, and glutamate in the brain and spinal cord, while the 0.25 mg/kg dose primarily reduced serotonin and NE levels in the spinal cord. Thus, the 0.25 mg/kg dose seems to exert a less toxic effect compared to the 0.5 mg/kg dose, accounting for the increased toxicity observed in tissues such as the thalamus and hippocampus following the higher dose which highlights the importance of monoamines in maintaining neural integrity. These findings suggest that the 0.5 mg/kg dose may be prioritised over the 0.25 mg/kg dose in long-duration studies (i.e., > 5 days). These findings have important therapeutic implications, particularly in developing interventions targeting monoamine depletion in FM and other neuropsychiatric disorders, and offer a foundation for future research focused on mitigating these adverse effects.

## Informed Consent Statement

Not applicable

## Institutional Review Board Statement

The entire study was conducted in strict adherence to the ethical standards set by the Biomedical Ethics Committee of King Abdulaziz University (Approval Number: 236–24). The research protocol was developed following the guidelines of the Animal Care and Use Committee (ACUC) at the Animal House Unit, King Fahd Medical Research Centre, which also approved all procedures involving animals.

## Funding

This project was funded by the 10.13039/501100011665Deanship of Scientific Research (DSR) at King Abdulaziz University, Jeddah, under grant no (GPIP: 1724-828-2024). The authors, therefore, acknowledge with thanks DSR for technical and financial support.

## CRediT authorship contribution statement

**Badrah S. Alghamdi:** Writing – review & editing, Supervision, Funding acquisition, Conceptualization. **Mervat M. Halawani:** Investigation. **Mona Ali Al-Thepyani:** Methodology. **Hani A. Alturkistani:** Methodology. **Hanin Abdulbaset AboTaleb:** Writing – original draft, Investigation, Formal analysis, Data curation, Conceptualization. **Emad A. Hindi:** Investigation. **Gamal S. Abd El-Aziz:** Investigation.

## Declaration of Competing Interest

The authors declare that they have no known competing financial interests or personal relationships that could have appeared to influence the work reported in this paper.

## Data Availability

The data will be available upon request.
