# Supplementary material for: Evaluation of reserpine-induced fibromyalgia in mice: A comparative behavioral, neurochemical, and histological assessment of two doses
Source: IBRO Neurosci Rep. 2024 Nov 9;19:1093–108. doi: 10.1016/j.ibneur.2024.11.002 (PMC12834026; doi:10.1016/j.ibneur.2024.11.002)
Supplement: Supplementary file 1 — Supplementary material [file mmc1.docx]

**6. Supplementary data**

In this study, we measured anxiety-like behaviour and memory in mice using different tests as described below:

1. **Central preference test (CPT)**

Rodents typically spend more time exploring the periphery of the arena in contact with the walls. Anxiolytic-like behaviour is indicated by mice spending more time exploring an unprotected central area (53). Central preference behaviour was assessed using an open field arena (45 cm × 45 cm × 15 cm) with an inner quadrant zone (25 cm × 25 cm) designated as the central area (52). The time spent in the central and peripheral zones was recorded for 3 minutes using the EthoVision system. The percentage of central-zone preference was calculated as

Central preference%= $\frac{Time in central zone}{Total experiment time} \times100$.

After each trial, faeces and urine were removed, and the surface was wiped with 70% ethanol. This test was performed on days 4 and 9 following the first reserpine injection.

1. **Elevated plus Maze (EPM)**

We used an EPM to assess anxiety-related behavior, a well-established paradigm for evaluating anxiety-like behavior in rodents [53]. The EPM apparatus consists of a central platform (6 cm × 6 cm) extended with two open arms (35 cm × 5 cm) and two closed arms (35 cm × 5 cm × 15 cm). Mice were individually placed in an open arm facing the center of the maze and allowed to move freely through the apparatus for 5 minutes [48]. The time spent in both arms was recorded by the EthoVision tracking XT8A system (Noldus Information Technology, Wageningen, The Netherlands), with the time spent in the open arms considered indicative of an anxiolytic profile.

1. **Y-maze test (Y-MT)**

The potential impact of various doses of reserpine on short-term spatial memory was assessed with the Y-MT (54). This test employs a Y-shaped apparatus comprising three symmetrical arms labelled A, B, and C, each measuring 10 cm wide and 60 cm high. At the beginning of the trial, each mouse was placed individually in the centre of the Y-maze and allowed to freely explore the arms for 5 minutes, during which the sequence of arm entries was recorded. An alternation was defined as a consecutive entry into three different arms without repetition (e.g., ABC, BAC, CBA, or ACB) (55). Spontaneous alternation was calculated using the following formula:

Spontaneous alternation (%) = $\frac{Number of altrnations}{Toal number of entrance}\times100$.

The Y-MT was performed on day 10 following the first reserpine injection.


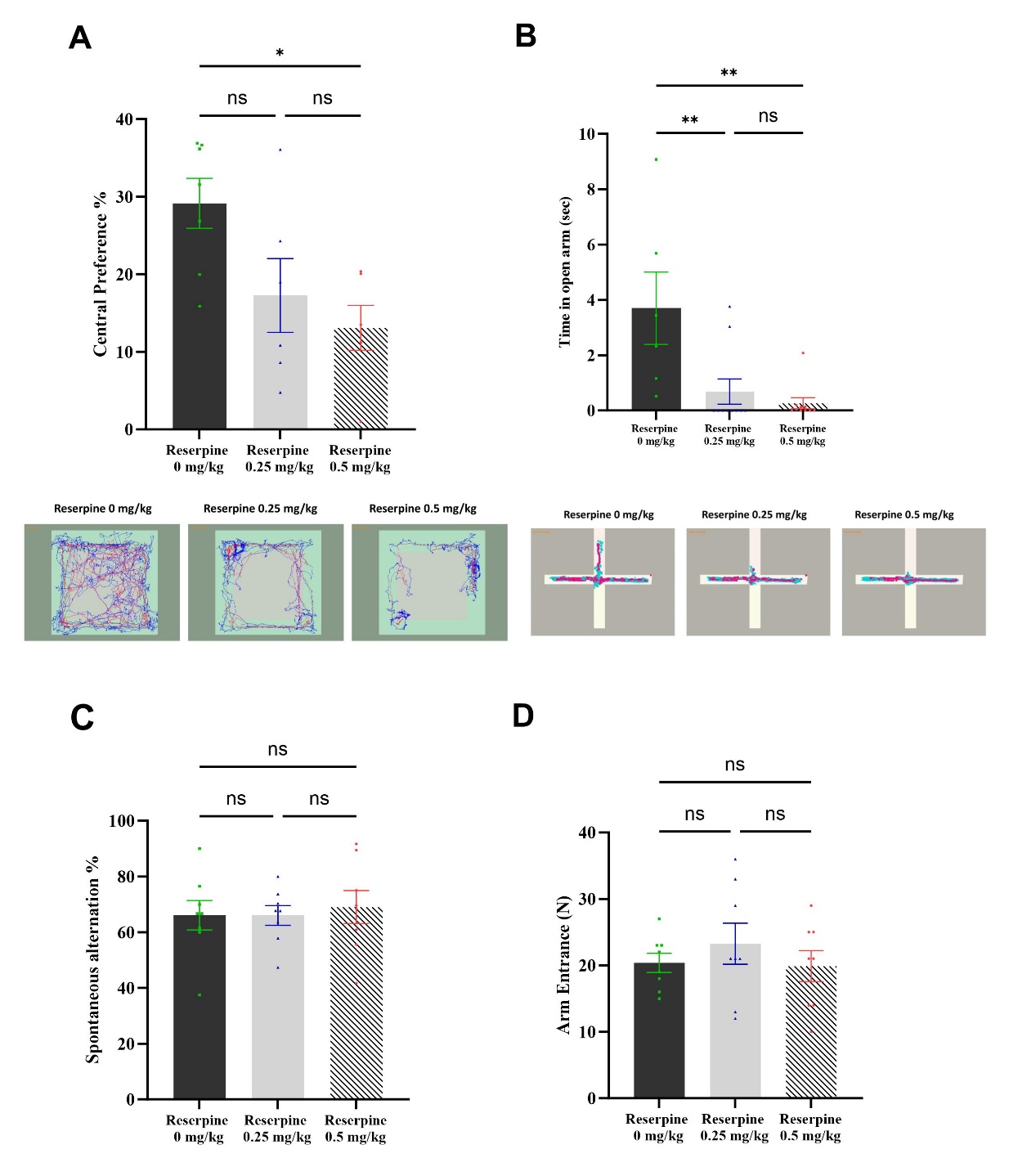


**Fig. 12. Supplementary**. Effect of both doses of reserpine on anxiety and memory tests. (A) Central preference test and track visualization of one mouse from each group during the test, (B) Elevated plus Maze test and track visualization of one mouse from each group during the test. (C) Spontaneous alteration in the Y-maze test, and (D) The number arm entrance in the Y-maze test. In the graphs, each bar represents the mean, and the vertical lines indicate the SEM of 6 - 8 mice/group. One-way ANOVA was used, followed by Tukey’s post-hoc test.
